# Hypertension and diabetes including their earlier stage are associated with increased risk of sudden cardiac arrest

**DOI:** 10.1038/s41598-022-16543-2

**Published:** 2022-07-19

**Authors:** Yun Gi Kim, Seung Young Roh, Kyung-Do Han, Joo Hee Jeong, Yun Young Choi, Kyongjin Min, Jaemin Shim, Jong-Il Choi, Young-Hoon Kim

**Affiliations:** 1 Division of Cardiology, Department of Internal Medicine, Korea University College of Medicine and Korea University Anam Hospital, 73 Goryeodae-ro, Seongbuk-gu, Seoul, 02841 Republic of Korea; 2 Division of Cardiology, Department of Internal Medicine, Korea University College of Medicine and Korea University Guro Hospital, Seoul, Republic of Korea; 3 Department of Statistics and Actuarial Science, Soongsil University, Seoul, Republic of Korea

**Keywords:** Cardiology, Risk factors

## Abstract

Sudden cardiac arrest (SCA) is a medical disaster for both the victim and the society. Despite intrinsic limitations in the management of SCA, primary prevention has been overlooked and risk factors for SCA are not fully understood. We aimed to evaluate whether hypertension and diabetes mellitus (DM), including pre-hypertension and impaired fasting glucose (IFG), are associated with increased risk of SCA. We performed a nationwide population-based analysis using the Korean National Health Insurance Service. People who underwent a national health check-up in 2009 were enrolled. The risk of SCA was evaluated in people with hypertension and DM with a clinical follow-up through December 2018. A total of 4,056,423 people with 33,345,378 person-years of follow-up and 16,352 SCA events were examined. People with hypertension had 65.4% increased risk of SCA (adjusted hazard ratio [HR] = 1.654 [1.572–1.739]; p < 0.001). Pre-hypertension was also associated with 21.3% increased risk of SCA (adjusted HR = 1.213 [1.158–1.272]; p < 0.001). People who had IFG and DM showed 7.5% (adjusted HR = 1.075 [1.035–1.117]; p < 0.001) and 80.1% (adjusted HR = 1.801 [1.731–1.875]; p < 0.001) increased risk of SCA, respectively. People with DM who took anti-diabetic medication showed significantly lower risk of SCA compared with uncontrolled DM patients (fasting glucose ≥ 200 mg/dL) (adjusted HR = 0.625 [0.533–0.733]; p < 0.001). Coexistence of hypertension and DM was associated with an even higher risk of SCA (adjusted HR = 3.078 [2.877–3.293]; p < 0.001). In conclusion, the risk of SCA is significantly higher in people with hypertension and DM, including pre-hypertension and IFG. Adequate control of blood pressure and serum glucose can have a profound impact for the primary prevention of SCA in the general population.

## Introduction

Sudden cardiac arrest (SCA) is an emergent medical condition with loss of mechanical contraction of the heart, and can lead to collapse in systemic circulation and death if left untreated^1^. To improve the survival of SCA patients, not only appropriate in-hospital management but also coordinated action of out-of-hospital management by the community is necessary^2,3^. Out-of-hospital management includes immediate recognition of cardiopulmonary arrest and initiation of cardiopulmonary resuscitation; activation of the emergency response system of the community; early defibrillation whenever possible; widespread utilization of automated defibrillators; and most importantly, high-quality education of the community members^3–8^. Although significant efforts have been made to improve survival of SCA patients, mortality is still high, with a recent study reporting 10.4% survival rate^9^.

Majority of SCA occurs outside of the hospital, and timely intervention can be difficult^3^. A significant proportion of SCA events are not witnessed, and education of the community members is labor-intensive and requires consistent long-term efforts^4,10^. Due to these obstacles and the intrinsic nature of SCA, a rapid progression to irreversible cardiac death, treatment of SCA after its occurrence is a difficult process^11^. Prior efforts have been focused on the treatment of SCA rather than its prevention^12^. Primary prevention of SCA can have a profound impact on public health considering its low survival rate and even lower probability of neurologically intact survival. Identifying risk factors for SCA should be the first step in preventing SCA. Because of the low incidence of SCA, the analysis of risk factors is limited using conventional cohorts. However, nationwide registries consisting of millions of people can enable such analysis.

Hypertension and diabetes mellitus (DM), which are recognized as atherosclerotic cardiovascular disease risk factors in the AHA/ACC guidelines, can increase the risk of SCA through their association with coronary artery disease, disease of the aorta, cerebrovascular accident, and heart failure^13,14^. However, whether pre-hypertension and impaired fasting glucose (IFG) can also increase the risk of SCA is not fully understood. Understanding the impact of hypertension and DM (including pre-hypertension and IFG) on SCA in the general population will have a profound impact on public health. We performed this nationwide population-based analysis to reveal the association between hypertension / DM (including pre-hypertension and IFG) and SCA.

## Methods

### Patients

We used the Korean National Health Insurance Service (K-NHIS) database to perform this study. The K-NHIS is an exclusive single insurance system, and all people of the Republic of Korea are mandatory subscribers. Therefore, medical data stored in the K-NHIS database can represent the entire Korean population. All subscribers of the K-NHIS have access to nationwide regular health screenings that include various laboratory tests such as blood cell count, renal function, liver function, fasting blood glucose (FBG), and lipid profile; measurements of blood pressure, height, and body weight; and self-questionnaires regarding alcohol consumption habits, smoking status, and physical activity level. Previous claim history of various International Classification of Disease, 10th revision (ICD-10) diagnostic codes such as hypertension, DM, or heart failure, and prescription history of various drugs are also available in the K-NHIS database.

Under the approval of study protocols by the relevant institutional review boards and the official review committee of the K-NHIS (https://nhiss.nhis.or.kr/), medical researchers are allowed to utilize the database of K-NHIS. The current study was approved by the Institutional Review Board of Korea University Medicine Anam Hospital and official review committee of the K-NHIS. Considering the retrospective nature of this study, the requirement for written informed consent was waived. The ethical guidelines of the 2013 Declaration of Helsinki and legal medical regulations of Republic of Korea were strictly undertaken throughout the study.

In this study, people who underwent a nationwide health screening in 2009 were enrolled. People with a diagnosis of SCA prior to enrollment or who were under 20 years of age were excluded. Data obtained from January 2002 to December 2008 were used to identity baseline medical history. Medical follow-up data were obtained until December 2018. There were no follow-up losses except for death and immigrations.

### Primary outcome endpoint

Occurrence of SCA was the main outcome of this study and both the aborted and non-aborted SCA was included. Sudden cardiac arrest was identified by claims of the following ICD-10 codes: I46.0 (cardiac arrest with successful resuscitation), I46.1 (sudden cardiac arrest), I46.9 (cardiac arrest, cause unspecified), I49.0 (ventricular fibrillation and flutter), R96.0 (instantaneous death), and R96.1 (death occurring less than 24 h from onset of symptoms). Only claims associated with declaration of death or cardiopulmonary resuscitation during an emergency department visit were identified as SCA events in this study. If participants of this study had a prior diagnosis of asphyxia, gastrointestinal bleeding, cerebral hemorrhage, ischemic stroke, sepsis, anaphylaxis, trauma, suffocation, hit by lightning, electric shock, drowning, or burn within six months of the diagnosis of SCA, the event was not counted as a primary outcome endpoint. The influence of hypertension and DM, including pre-hypertension and IFG, was evaluated. The incidence of SCA was defined as event numbers per 1,000 person-years of follow-up.

### Definitions

Hypertension was classified into four stages in this study: (i) non-hypertension (people with no prior diagnosis of hypertension; systolic blood pressure [SBP] < 120 and diastolic blood pressure [DBP] < 80); (ii) pre-hypertension (people with no prior diagnosis of hypertension; SBP 120–140 or DBP 80–90); (iii) hypertension without medication (people with a prior diagnosis of hypertension; SBP ≥ 140 or DBP ≥ 90, but not taking medication); and (iv) hypertension with medication (people diagnosed with and taking medication for hypertension). Only type 2 DM was analyzed in this study, and patients were classified into 5 stages: (i) non-DM (FBG < 100 mg/dl and absence of diagnosis by physician and medications for DM); (ii) IFG (FBG ranging from 100 to 125 mg/dl and absence of diagnosis by physician and medications for DM); (iii) new-onset DM (FBG ≥ 126 mg/dl and absence of diagnosis by physician and medications for DM); (iv) DM for less than 5 years (FBG ≥ 126 mg/dl or physician diagnosis of DM within 5 years; and (v) DM for more than 5 years (FBG ≥ 126 mg/dl or physician diagnosis of DM for more than 5 years). Our prior studies have demonstrated the robustness of these classifications^15–18^.

### Statistical analysis

Student’s t-test was used to compare continuous variables. Chi-square test or Fisher’s exact test was performed to compare categorical variables whenever appropriate. Kaplan–Meier curve analysis was conducted to depict the cumulative incidence of SCA, and between-group differences were compared using the log-rank t-test. Non-adjusted and adjusted hazard ratios (HRs) and their 95% confidence intervals (CIs) were calculated using Cox regression analysis. In the multivariate model, age, sex, body mass index (BMI), waist circumference, income level, smoking status, alcohol consumption status, regular physical activity, hypertension, DM, dyslipidemia, chronic kidney disease, and I-glutamyl transferase were adjusted. Death was additionally adjusted as a competing risk. All tests were two-tailed, and p values ≤ 0.05 were considered statistically significant. All statistical analyses were performed with SAS version 9.2 (SAS Institute, Cary, NC, USA).

### Ethics approval

The current study was approved by the Institutional Review Board of Korea University Medicine Anam Hospital and official review committee of the K-NHIS. Considering the retrospective nature of this study, the requirement for written informed consent was waived. The ethical guidelines of the 2013 Declaration of Helsinki and legal medical regulations of Republic of Korea were strictly undertaken throughout the study.

## Results

### Patients

**Figure 1:**
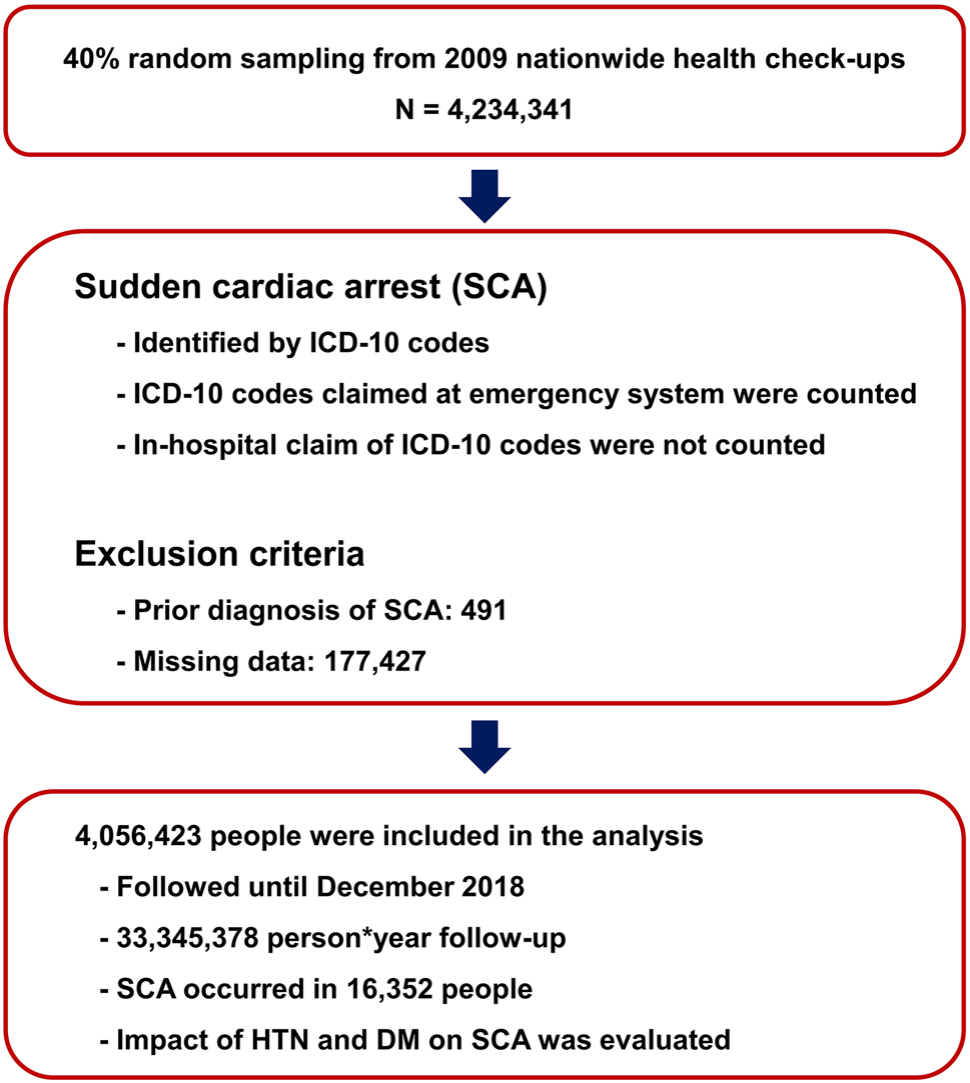
Study flow. *DM* diabetes mellitus, *HTN* hypertension; *ICD-10* international classification of disease, 10th revision, *SCA* sudden cardiac arrest.

In 2009, 66% of people who were supposed to undergo nationwide health check-up actually underwent health check-up. Among people older than 20 years who underwent a nationwide health screening in 2009, 50% of them were randomly selected in this analysis and a total of 4,234,341 people were enrolled. Among this group, 491 and 177,427 people were excluded due to prior diagnosis of SCA and missing data, respectively. The flow of this study is summarized in Fig. 1. During 33,345,378 person-years of follow-up, 16,352 SCA events occurred (incidence per 1000 person-years = 0.490). Baseline demographics between people who did and did not experience SCA during the follow-up period are summarized in Table 1. In brief, people who had an SCA event were more likely to be male, older, and current smokers; had a higher prevalence of hypertension, DM, chronic kidney disease (CKD; estimated glomerular filtration rate < 60 ml/min/1.73m^2^), and dyslipidemia; and had higher SBP, DBP, and FBG. Waist circumference was significantly higher in people who experienced SCA, but BMI did not differ significantly between the two groups.

**Table 1:** Baseline characteristics of patients with and without SCA.

	SCA		p-value
	No	Yes	
	4,040,071	16,352	
Male	2,221,898 (55.0%)	11,633 (71.1%)	< 0.001
Age (years)	47.0 ± 14.1	62.0 ± 13.2	< 0.001
**Age group**			
20–29	501,371 (12.4%)	245 (1.5%)	< 0.001
30–39	778,093 (19.3%)	717 (4.4%)	
40–49	1,063,833 (26.3%)	2026 (12.4%)	
50–59	857,767 (21.2%)	3181 (19.5%)	
60–69	543,973 (13.5%)	4515 (27.6%)	
70–79	257,092 (6.4%)	4591 (28.1%)	
80	37,942 (0.9%)	1077 (6.6%)	
Body mass index (kg/m^2^)	23.7 ± 3.2	23.8 ± 3.4	0.138
Waist circumference (cm)	80.2 ± 9.5	83.5 ± 8.9	< 0.001
**Smoking**			
Never-smoker	2,399,679 (59.4%)	7916 (48.4%)	< 0.001
Ex-smoker	581,485 (14.4%)	3128 (19.1%)	
Current-smoker	1,058,907 (26.2%)	5308 (32.5%)	
**Alcohol consumption**			
Non-drinker	2,077,053 (51.4%)	9534 (58.3%)	< 0.001
Mild-drinker	1,641,427 (40.6%)	5263 (32.2%)	
Heavy-drinker	321,591 (8.0%)	1555 (9.5%)	
Regular exercise	733,609 (18.2%)	3148 (19.3%)	< 0.001
Income (lowest 20%)	704,587 (17.4%)	3075 (18.8%)	< 0.001
Diabetes mellitus	349,134 (8.6%)	4264 (26.1%)	< 0.001
**Diabetes mellitus stage**			
Non-diabetic	2,776,161 (68.72%)	7970 (48.7%)	< 0.001
Impaired fasting glucose	914,776 (22.64%)	4118 (25.2%)	
New onset diabetes	119,558 (2.96%)	1025 (6.3%)	
Diabetic < 5 years	118,215 (2.93%)	1272 (7.8%)	
Diabetic ≥ 5 years	111,361 (2.76%)	1967 (12.0%)	
Glucose (mg/dL)	97.2 ± 23.8	110.0 ± 41.5	< 0.001
Hypertension	1,082,382 (26.8%)	9331 (57.1%)	< 0.001
**Hypertension stage**			
Non-hypertensive	1,383,411 (34.24%)	2566 (15.7%)	< 0.001
Pre-hypertension	1,574,278 (38.97%)	4455 (27.2%)	
Hypertension	334,302 (8.27%)	1777 (10.9%)	
Hypertension with medication	748,080 (18.52%)	7554 (46.2%)	
Systolic blood pressure (mmHg)	122.4 ± 15.0	129.3 ± 17.2	< 0.001
Diastolic blood pressure (mmHg)	76.3 ± 10.0	78.9 ± 11.0	< 0.001
Dyslipidemia	732,983 (18.1%)	4610 (28.2%)	< 0.001
**Dyslipidemia stage**			
Total cholesterol < 240 (mg/dL)	3,307,088 (81.9%)	11,742 (71.8%)	< 0.001
Total cholesterol ≥ 240	347,131 (8.6%)	1541 (9.4%)	
Total cholesterol ≥ 240 with medication	385,852 (9.6%)	3069 (18.8%)	
Cholesterol (mg/dL)	195.3 ± 41.1	195.1 ± 44.3	0.549
High-density lipoprotein (mg/dL)	56.5 ± 32.9	53.6 ± 30.9	< 0.001
Low-density lipoprotein (mg/dL)	121.2 ± 214.2	115.0 ± 97.8	< 0.001
Chronic kidney disease	275,854 (6.8%)	2740 (16.8%)	< 0.001
eGFR (mL/min/1.73m^2^)	87.6 ± 44.9	80.4 ± 34.7	< 0.001

*eGFR* estimated glomerular filtration rate, *SCA* sudden cardiac arrest.

### Hypertension

**Figure 2:**
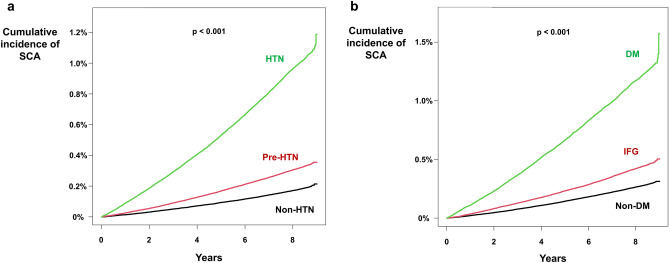
Impact of hypertension and diabetes mellitus on SCA. **(a)** Kaplan–Meier curve analysis showed a significantly higher cumulative incidence of SCA in the hypertension and pre-hypertension groups. (**b**) Diabetes mellitus and IFG were associated with a significantly higher cumulative incidence of SCA. *DM* diabetes mellitus, *HTN* hypertension, *IFG* impaired fasting glucose, *SCA* sudden cardiac arrest.

Sudden cardiac arrest occurred in 2566 (incidence = 0.223), 6232 (incidence = 0.395), and 7554 (incidence = 1.243) people in non-hypertension, pre-hypertension, and hypertension group, respectively. The risk of SCA was higher in the pre-hypertension group (HR = 1.770 [1.690–1.853]; p < 0.001) and hypertension group (HR = 5.565 [5.321–5.820]; p < 0.001). Kaplan–Meier curve analysis showed significantly higher cumulative incidence of SCA in the pre-hypertension and hypertension groups as compared with the non-hypertension group (p < 0.001; Fig. 2a).

People with pre-hypertension and hypertension showed significantly different baseline clinical characteristics compared with the non-hypertension group (Supplementary Table S1). After adjusting differences in baseline demographics, people with pre-hypertension and hypertension had 21.3% (HR = 1.213 [1.158–1.272]; p < 0.001; Table 2) and 65.4% (HR = 1.654 [1.572–1.739]; p < 0.001; Table 2) increased risk of SCA, respectively.

**Table 2:** Impact of hypertension and diabetes mellitus on SCA.

	n	SCA	Follow-up duration (person-years)	Incidence	Hazard ratio with 95% confidence interval		
					Univariate	Multivariate 1	Multivariate 2
**Diabetes mellitus**							
Non-diabetic	2,784,131	7970	22,996,956	0.347	1 (reference)	1 (reference)	1 (reference)
IFG	918,894	4118	7,533,750	0.547	1.579 (1.520–1.639)	1.075 (1.035–1.117)	1.075 (1.034–1.117)
Diabetes Mellitus	353,398	4264	2,814,672	1.515	4.382 (4.222–4.549)	1.801 (1.731–1.875)	1.732 (1.663–1.803)
**Hypertension**							
Non-hypertensive	1,385,977	2566	11,491,069	0.223	1 (reference)	1 (reference)	1 (reference)
Pre-hypertension	1,914,812	6232	15,777,568	0.395	1.770 (1.690–1.853)	1.213 (1.158–1.272)	1.213 (1.157–1.272)
Hypertension	755,634	7554	6,076,741	1.243	5.565 (5.321–5.820)	1.654 (1.572–1.739)	1.629 (1.547–1.715)

Incidence is per 1000 person-years follow-up.

Multivariate model 1 is adjusted for age, sex, body mass index, waist circumference, smoking status, alcohol consumption, regular physical activity, income level, ɣ-glutamyl transferase, hypertension, diabetes mellitus, dyslipidemia, and chronic kidney disease.

Multivariate model 2 if further adjusted for death as a competing risk.

*IFG* impaired fasting glucose, *SCA* sudden cardiac arrest.

### Diabetes

Among study population, 7970 (incidence = 0.347), 4118 (incidence = 0.547), and 4264 (incidence = 1.515) people experienced SCA in non-DM, IFG, and DM group, respectively. The risk of SCA was significantly higher in people with IFG (HR = 1.579 [1.520–1.639]; p < 0.001; Table 2) and DM (HR = 4.382 [4.222–4.549]; p < 0.001; Table 2) compared with the non-DM group. Cumulative incidence of SCA was also significantly higher in people with IFG and DM in Kaplan–Meier curve analysis (Fig. 2b). After adjustment of covariates which were significantly different according to diabetic stage, IFG and DM were associated with 7.5% (HR = 1.075 [1.035–1.117]; p < 0.001; Table 2) and 80.1% (HR = 1.801 [1.731–1.875]; p < 0.001; Table 2) increased risk of SCA, respectively, compared with the non-DM group.

Fasting blood glucose level showed a significant association with the risk of SCA. As compared with people with normal fasting glucose, people with IFG showed a significantly higher risk of SCA (HR = 1.070 [1.030–1.112]; p < 0.001; Table 3). People with a fasting blood glucose level of 126–200 (HR = 1.494 [1.391–1.605]; p < 0.001; Table 3) and ≥ 200 (HR = 2.981 [2.551–3.484]; p < 0.001; Table 3) had a significantly increased risk of SCA. People who were taking diabetic medication demonstrated a significantly higher risk of SCA compared to people with normal fasting glucose (HR = 1.863 [1.782–1.947]; p < 0.001; Table 3) but a lower risk compared to people with fasting blood glucose ≥ 200 (HR = 0.625 [0.533–0.733]; p < 0.001).

**Table 3:** Risk of SCA according to fasting blood glucose level.

	n	SCA	Follow-up duration (person-years)	Incidence	Hazard ratio with 95% confidence interval	
					Univariate	Multivariate
**Fasting glucose level**						
60–100	2,784,131	7970	22,996,956	0.347	1 (reference)	1 (reference)
100–126	918,894	4118	7,533,750	0.547	1.579 (1.520–1.639)	1.070 (1.030–1.112)
126–200	108,379	862	875,184	0.985	2.852 (2.658–3.059)	1.494 (1.391–1.605)
≥ 200	12,204	163	96,655	1.686	4.913 (4.208–5.738)	2.981 (2.551–3.484)
On diabetic medication	232,815	3239	1,842,833	1.758	5.081 (4.878–5.293)	1.863 (1.782–1.947)

Incidence is per 1,000 person-years of follow-up.

Multivariate model is adjusted for age, sex, body mass index, waist circumference, smoking status, alcohol consumption, regular physical activity, income level, ɣ-glutamyl transferase, hypertension, diabetes mellitus, dyslipidemia, and chronic kidney disease.

*SCA* sudden cardiac arrest.

**Figure 3:**
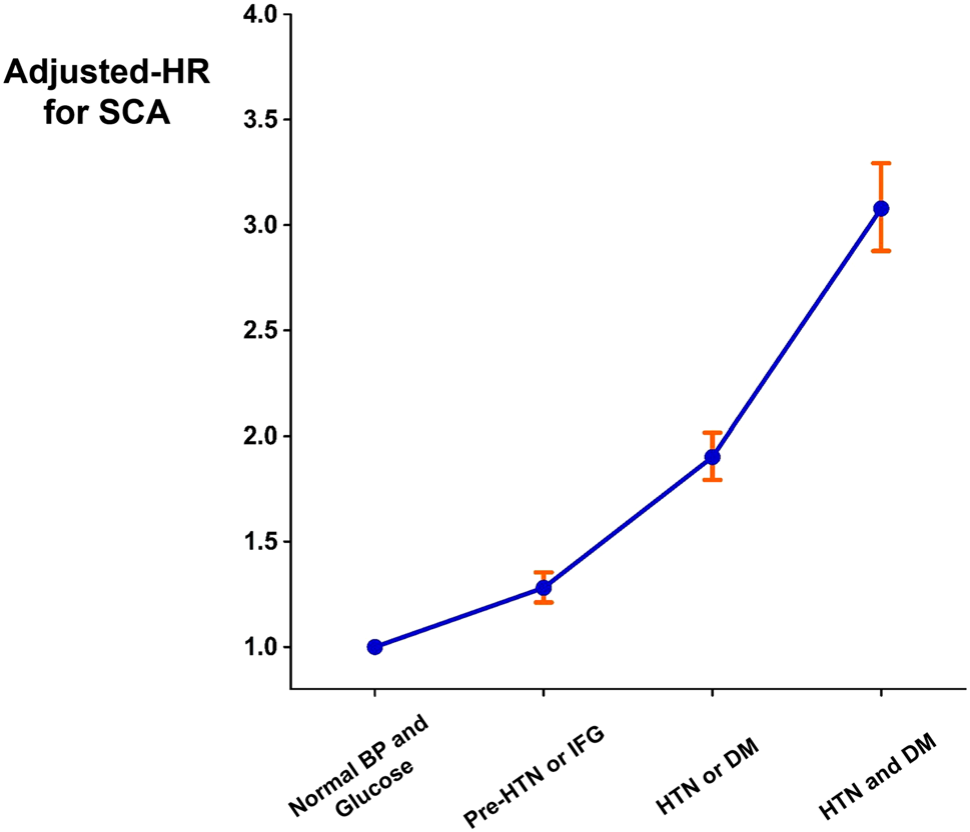
Risk of SCA in people with both hypertension and DM. People with pre-HTN, IFG, hypertension, or DM showed significantly increased risk of SCA as compared with the normal control group. However, the risk was greatest in people with both hypertension and DM, suggesting a synergistic effect with both diseases. *BP* blood pressure, *DM* diabetes mellitus, *HTN* hypertension, *IFG* impaired fasting glucose, *SCA* sudden cardiac arrest.

People who had either pre-hypertension or IFG had 28.1% increased risk of SCA compared with people who had normal blood pressure and euglycemia (HR = 1.281 [1.211–1.354]; p < 0.001; Table 4; Fig. 3). People with either hypertension or DM demonstrated 90.1% increased risk of SCA (HR = 1.901 [1.793–2.017]; p < 0.001; Table 4; Fig. 3). The risk of SCA was 3.078-fold higher if both hypertension and DM were present (HR = 3.078 [2.877–3.293]; p < 0.001; Table 4; Fig. 3).

**Table 4:** Combined effect of hypertension and diabetes mellitus on SCA.

Hypertension and DM status	n	SCA	Follow-up duration (person-years)	Incidence	Hazard ratio with 95% confidence interval	
					Univariate	Multivariate
No HTN, DM, pre-HTN, and IFG	1,109,597	1,645	9,214,601	0.179	1 (reference)	1 (reference)
Either pre-HTN or IFG	2,015,257	5,725	16,632,822	0.344	1.930 (1.827–2.038)	1.281 (1.211–1.354)
Either HTN or DM	754,106	6,146	6,104,496	1.007	5.640 (5.341–5.956)	1.901 (1.793–2.017)
Both HTN and DM	177,463	2,836	1,393,458	2.035	11.445 (10.770–12.162)	3.078 (2.877–3.293)

Incidence is per 1000 person-years of follow-up.

Multivariate model is adjusted for age, sex, body mass index, waist circumference, smoking status, alcohol consumption, regular physical activity, income level, ɣ-glutamyl transferase, hypertension, diabetes mellitus, dyslipidemia, and chronic kidney disease.

*DM* diabetes mellitus, *HTN* hypertension, *IFG* impaired fasting glucose, *SCA* sudden cardiac arrest.

## Discussion

The main findings of this study can be summarized as follows: (i) hypertension and DM are significantly associated with an increased risk of SCA; (ii) the risk of SCA is also increased in people with pre-hypertension and IFG; (iii) uncontrolled DM had a significantly higher risk of SCA compared with DM patients taking medications; and (iv) people with both hypertension and DM had an increased risk of SCA as compared with people having only one of those conditions. Despite the grave prognosis and devastating socioeconomic impact of SCA, its relatively low incidence was an obstacle for identification of risk factors for SCA. By utilizing a nationwide population-based cohort, we demonstrated that hypertension and DM, including their earlier stages of disease, are important risk factors for SCA.

### Primary prevention

The chance for survival is very low once cardiopulmonary arrest occurs outside the hospital and is even lower for neurologically intact survival^19–21^. Since high-quality education and training of the general population is difficult to achieve and a disseminated supply of automated defibrillators is not feasible in most of the world, treatment of SCA is fundamentally limited. The clinical outcome of SCA is disappointing even in highly organized societies such as Seattle, Washington, USA^22,23^. Due to the intrinsic nature of SCA and associated grave prognosis, primary prevention of SCA should be emphasized even if a given society is highly trained and well equipped with automated defibrillators.

Identification of risk factors for SCA is the first step to prevent SCA. However, due to the relatively low incidence of SCA, not much is known about the risk factors of SCA. In 2005, Thorgeirsson et al. reported that established coronary artery disease, current smoking, high blood pressure, and hypercholesterolemia were associated with SCA^24^. However, the study was limited by low sample size (SCA events in 137 of the 8006 men and 44 of the 9435 women) and geographically limited area (Reykjavik). Although hypertension was shown to be associated with SCA in both Reykjavik and the current study, DM was associated with SCA only in this study. Our study also demonstrated a graded risk of SCA in people with non-hypertension / pre-hypertension / hypertension and non-DM / IFG / DM. We observed 65.4% and 80.1% increased risk of SCA in people with hypertension and DM, respectively. Furthermore, the co-presence of hypertension and DM was associated with a more than a threefold increased risk of SCA, indicating a synergistic effect between hypertension and DM. Our results indicate the usefulness of hypertension and DM for the risk prediction of SCA and suggest the importance of managing these most widespread diseases in the modern era.

In this study, people with uncontrolled DM (fasting serum glucose ≥ 200 mg/dL) showed a nearly threefold increased risk of having SCA compared with people maintaining a normal fasting glucose level (Table 3). They also had a higher risk of SCA than DM patients taking anti-diabetic medications, suggesting that appropriate treatment of DM might reduce the risk of SCA. Whether sequential decrease in blood pressure and fasting glucose will lead to decreased risk of SCA will be an important area of future research.

### Pre-hypertension and IFG

The impact of pre-hypertension and IFG on SCA has been poorly understood. In this analysis, there was a clear increase in the risk of SCA in people with pre-hypertension and IFG, suggesting that these patient groups might benefit from strict lifestyle modification or medical treatment. Recently, we reported that pre-hypertension and IFG are associated with a significantly increased risk of atrial fibrillation^17,18^. Evidence supporting medical treatment of pre-hypertension and IFG is lacking, and these conditions are usually left untreated in real world clinical practice. However, our results suggest that lifestyle modifications, if not medical treatment, will benefit people with pre-hypertension and IFG with respect to SCA. Pre-hypertension and IFG can be considered as a risk factor for SCA, according to our analysis.

Blood pressure and serum glucose are continuous variables, and a specific cut-off value to define a high-risk group for a certain disease might not be feasible. They can be considered as a continuum, and risk of medical disease might gradually increase as blood pressure or serum glucose increases, as demonstrated in our prior study for AF and in this study for SCA^17,18^.

### Study limitations

Several limitations exist in this study. First, coding inaccuracies might exist in this study since it was based on retrospective analysis of data stored in a nationwide health insurance organization. Since diagnosis of SCA was based on claim of ICD-10 codes, sophisticated adjudication of SCA, especially through autopsy, was not possible. However, multiple prior publications have validated our coding strategies for various medical conditions such as hypertension and DM^15–18,25^. Second, our analysis focused on the occurrence of SCA; the clinical course of SCA events was not available, and type of treatment performed for SCA events could not be analyzed. Third, the current study is solely based on East Asian people, therefore, extrapolation of our results to different ethnic groups should be done with caution. Although virtually all people in South Korea are mandatory subscribers of the K-NHIS which recommends to take regular health check-ups, some people do not participate in the health check-up program. Therefore, our cohort can have some different demographics compared with the entire people of South Korea. Fourth, whether medical treatments for hypertension and DM will reduce the risk of SCA needs further evaluation. Fifth, the impact of type 1 DM on SCA were not evaluated in this study.

## Conclusions

The risk of SCA is significantly increased in people with hypertension and DM. The risk was higher if hypertension and DM coexisted. People with pre-hypertension and IFG also had a significantly elevated risk of SCA. Efforts to control blood pressure and serum glucose can have a significant influence on public health with respect to the prevention of SCA.

## Supplementary Information

Supplementary Table S1.

## Data Availability

The data underlying this article are available in the article and in its online supplementary material.

## Abbreviations

BMI: Body mass index
CI: Confidence interval
CKD: Chronic kidney disease
DBP: Diastolic blood pressure
DM: Diabetes mellitus
FBG: Fasting blood glucose
HR: Hazard ratio
ICD: International Classification of Disease
IFG: Impaired fasting glucose
K-NHIS: Korean National Health Insurance Service
SCA: Sudden cardiac arrest
SBP: Systolic blood pressure
