# Supplementary material for: Hypertension and diabetes including their earlier stage are associated with increased risk of sudden cardiac arrest
Source: Sci Rep. 2022 Jul 19;12:12307. doi: 10.1038/s41598-022-16543-2 (PMC9296606; doi:10.1038/s41598-022-16543-2)
Supplement: Supplementary file 1 — Supplementary Table S1. [file 41598_2022_16543_MOESM1_ESM.docx]

**Hypertension and Diabetes Including Their Earlier Stage are Associated with Increased Risk of Sudden Cardiac Death**

Yun Gi Kim,^1^ Seung Young Roh,^2^ Kyung-Do Han,^3^ Joo Hee Jeong,^1^ Subin Lim,^1^ Yun Young Choi,^1^ Kyongjin Min,^1^ Jaemin Shim,^1^ Jong-Il Choi,^1^* and Young-Hoon Kim^1^

^1^Division of Cardiology, Department of Internal Medicine, Korea University College of Medicine and Korea University Anam Hospital, Seoul, Republic of Korea

^2^Division of Cardiology, Department of Internal Medicine, Korea University College of Medicine and Korea University Guro Hospital, Seoul, Republic of Korea

^3^Department of Statistics and Actuarial Science, Soongsil University, Seoul, Republic of Korea

*Address for correspondence: Jong-Il Choi, MD, PhD, MHSc

^1^Division of Cardiology, Department of Internal Medicine, Korea University College of Medicine and Korea University Anam Hospital, Seoul, Republic of Korea

73 Goryeodae-ro, Seongbuk-gu, Seoul 02841, Republic of Korea

Tel: 82-2-920-5445

Fax: 82-2-927-1478

E-mail: [jongilchoi@korea.ac.kr](mailto:jongilchoi@korea.ac.kr)

**Running title:** Hypertension, diabetes, and sudden cardiac death

**Disclosure:** The authors have nothing to disclose.

**Total word count:** 5,305

The first two authors contributed equally to this work.

**Supplementary Table S1.** Baseline characteristics of patients with and without SCA.

|  | **SCA** | | **p-value** |
| --- | --- | --- | --- |
|  | **No** | **Yes** |  |
|  | 4,040,071 | 16,352 |  |
| **Male** | 2,221,898 (55.0%) | 11,633 (71.1%) | < 0.001 |
| **Age (years)** | 47.0 ± 14.1 | 62.0 ± 13.2 | < 0.001 |
| **Age group** |  |  | < 0.001 |
| 20 – 29 | 501,371 (12.4%) | 245 (1.5%) |  |
| 30 – 39 | 778,093 (19.3%) | 717 (4.4%) |  |
| 40 – 49 | 1,063,833 (26.3%) | 2,026 (12.4%) |  |
| 50 – 59 | 857,767 (21.2%) | 3,181 (19.5%) |  |
| 60 – 69 | 543,973 (13.5%) | 4,515 (27.6%) |  |
| 70 – 79 | 257,092 (6.4%) | 4,591 (28.1%) |  |
| 80 – | 37,942 (0.9%) | 1,077 (6.6%) |  |
| **Body mass index (kg/m^2^)** | 23.7 ± 3.2 | 23.8 ± 3.4 | 0.138 |
| **Waist circumference (cm)** | 80.2 ± 9.5 | 83.5 ± 8.9 | < 0.001 |
| **Smoking** |  |  | < 0.001 |
| Never-smoker | 2,399,679 (59.4%) | 7,916 (48.4%) |  |
| Ex-smoker | 581,485 (14.4%) | 3,128 (19.1%) |  |
| Current-smoker | 1,058,907 (26.2%) | 5,308 (32.5%) |  |
| **Alcohol consumption** |  |  | < 0.001 |
| Non-drinker | 2,077,053 (51.4%) | 9,534 (58.3%) |  |
| Mild-drinker | 1,641,427 (40.6%) | 5,263 (32.2%) |  |
| Heavy-drinker | 321,591 (8.0%) | 1,555 (9.5%) |  |
| **Regular exercise** | 733,609 (18.2%) | 3,148 (19.3%) | < 0.001 |
| **Income (lowest 20%)** | 704,587 (17.4%) | 3,075 (18.8%) | < 0.001 |
| **Diabetes mellitus** | 349,134 (8.6%) | 4,264 (26.1%) | < 0.001 |
| **Diabetes mellitus stage** |  |  | < 0.001 |
| Non-diabetic | 2,776,161 (68.72%) | 7,970 (48.7%) |  |
| Impaired fasting glucose | 914,776 (22.64%) | 4,118 (25.2%) |  |
| New onset diabetes | 119,558 (2.96%) | 1,025 (6.3%) |  |
| Diabetic < 5 years | 118,215 (2.93%) | 1,272 (7.8%) |  |
| Diabetic ≥ 5 years | 111,361 (2.76%) | 1,967 (12.0%) |  |
| **Glucose (mg/dL)** | 97.2 ± 23.8 | 110.0 ± 41.5 | < 0.001 |
| **Hypertension** | 1,082,382 (26.8%) | 9,331 (57.1%) | < 0.001 |
| **Hypertension stage** |  |  | < 0.001 |
| Non-hypertensive | 1,383,411 (34.24%) | 2,566 (15.7%) |  |
| Pre-hypertension | 1,574,278 (38.97%) | 4,455 (27.2%) |  |
| Hypertension | 334,302 (8.27%) | 1,777 (10.9%) |  |
| Hypertension with medication | 748,080 (18.52%) | 7,554 (46.2%) |  |
| **Systolic blood pressure (mmHg)** | 122.4 ± 15.0 | 129.3 ± 17.2 | < 0.001 |
| **Diastolic blood pressure (mmHg)** | 76.3 ± 10.0 | 78.9 ± 11.0 | < 0.001 |
| **Dyslipidemia** | 732,983 (18.1%) | 4,610 (28.2%) | < 0.001 |
| **Dyslipidemia stage** |  |  | < 0.001 |
| Total cholesterol < 240 (mg/dL) | 3,307,088 (81.9%) | 11,742 (71.8%) |  |
| Total cholesterol ≥ 240 | 347,131 (8.6%) | 1,541 (9.4%) |  |
| Total cholesterol ≥ 240 with medication | 385,852 (9.6%) | 3,069 (18.8%) |  |
| **Cholesterol (mg/dL)** | 195.3 ± 41.1 | 195.1 ± 44.3 | 0.549 |
| **High-density lipoprotein (mg/dL)** | 56.5 ± 32.9 | 53.6 ± 30.9 | < 0.001 |
| **Low-density lipoprotein (mg/dL)** | 121.2 ± 214.2 | 115.0 ± 97.8 | < 0.001 |
| **Chronic kidney disease** | 275,854 (6.8%) | 2,740 (16.8%) | < 0.001 |
| **eGFR (mL/min/1.73m^2^)** | 87.6 ± 44.9 | 80.4 ± 34.7 | < 0.001 |

eGFR: estimated glomerular filtration rate; SCA: sudden cardiac arrest.
